# Serum immune profiling suggests overlap between IBD patients with joint complaints and patients with spondyloarthritis

**DOI:** 10.3389/fimmu.2026.1827501

**Published:** 2026-07-09

**Authors:** Suzanne H. C. Veltkamp, Floris A. van Gaalen, Liese J. E. de Bruin, Sanne J. van Erp, Janneke N. Samsom, Andrea E. van der Meulen-de Jong, Philip W. Voorneveld

**Affiliations:** 1 Department of Gastroenterology and Hepatology, Leiden University Medical Center (LUMC), Leiden, Netherlands; 2 Department of Rheumatology, Leiden University Medical Center (LUMC), Leiden, Netherlands; 3 Department of Gastroenterology and Hepatology, St. Antonius Hospital Nieuwegein, Nieuwegein, Netherlands; 4 Laboratory of Pediatrics – div. Gastroenterology and Nutrition, Erasmus University Medical Center – Sophia Children’s Hospital, Rotterdam, Netherlands

**Keywords:** chronic inflammation, gut-joint axis, inflammatory bowel disease, serum immune profile, spondyloarthritis

## Abstract

**Background:**

Inflammatory bowel disease (IBD) and spondyloarthritis (SpA) are strongly associated, and joint complaints are common in patients with IBD. However, it remains unclear whether joint complaints in IBD merely reflect non-specific symptoms, or whether they are associated with systemic immune features overlapping with SpA.

**Methods:**

Using a proximity extension assay, 92 inflammation-related proteins were quantified in serum samples of patients from two cohorts: (i) IBD patients without (n = 100) and with joint complaints (n = 155; of whom 15 with SpA), and (ii) 169 patients with chronic back pain of unknown origin at the rheumatology outpatient clinic, where diagnosis of SpA was confirmed (n = 85) or rejected after 2 years of follow-up. Serum samples were collected at baseline.

**Results:**

IBD patients without joint complaints from cohort (i) and confirmed axSpA patients from cohort (ii) showed distinct serum immune profiles, with 20 differentially abundant proteins. Among IBD patients with joint complaints, formal SpA diagnosis had limited additional impact on the serum immune profile, with only three differentially abundant proteins between those with and without SpA. Similarly, among patients with chronic back pain, serum immune profiles were largely concordant irrespective of subsequent axSpA diagnosis. Importantly, IBD patients with joint complaints differed from IBD patients without joint complaints, and several proteins increased in this comparison overlapped with proteins increased in axSpA.

**Conclusions:**

Joint complaints in IBD are associated with a modest but detectable shift in the circulating immune profile, partly overlapping with axSpA-associated proteins. In contrast, formal SpA diagnosis adds limited additional serum-protein separation among patients who already have joint symptoms. These findings suggest that joint complaints in IBD may mark a biologically relevant subgroup beyond current SpA classification, and support longitudinal studies to determine whether this subgroup has distinct clinical trajectories or treatment responses.

## 1 Introduction

Inflammatory bowel disease (IBD) is strongly associated with joint complaints (JC), as demonstrated by the high prevalence (~30%) of musculoskeletal manifestations in IBD patients (1). Among these manifestations, arthralgia is the most common diagnosis, with a prevalence of 14.3-22% in Crohn’s disease (CD) patients, and a prevalence of 5.3-14% in ulcerative colitis (UC) patients (2, 3). Additionally, spondyloarthritis (SpA) has a substantially higher prevalence in IBD patients (up to 13%) compared to the general population (0.2-1.6%) (4, 5). IBD and SpA are both immune-mediated inflammatory disorders (IMIDs), and they share many features, including genetic risk factors (e.g. IL23R), changes in the gut microbiota (e.g. increased *R. gnavus* abundance), and therapeutic options targeting e.g. TNF-α, IL-23, or JAK (6). Despite available therapies for both diseases, chronic inflammation persists in many patients, and only 50-70% initially respond to treatment (7–9). Disease heterogeneity likely contributes to this challenge (10, 11).

Despite these shared features, there are notable differences in the pathogenesis of IBD and SpA. For example, IL-17A plays a role in the pathogenesis of and is a drug target for SpA, but has a protective role in IBD (12). In addition, nonsteroidal anti-inflammatory drugs are commonly used in SpA treatment, but are contra-indicated for IBD (13). Vedolizumab, a drug available for IBD patients targeting the α4β7 integrin, has been linked to the occurrence of arthralgia in some IBD patients (14). The differences in treatment effects on the gut versus on joints are also indicative of differences in pathogenesis between IBD and SpA. As a result, IBD-SpA is often managed as two separate conditions, and no clinical trials have specifically investigated the effects of immunomodulatory drugs specifically in patients with both IBD and SpA.

There are many different clinical criteria to classify SpA patients, including the Assessment of SpondyloArthritis international Society (ASAS), Amor, and European Spondyloarthropathy Study Group (ESSG) criteria. These criteria differ in their requirements, such as the need for radiological evidence, the use of magnetic resonance imaging (MRI), and the distinction between peripheral and axial SpA. In studies investigating the effects of treatment on extraintestinal manifestations in IBD, the definitions used for arthritis are heterogeneous, while studies investigating treatment effects on extra-articular outcomes in SpA patients are still lacking (15). The limited available studies show that anti-TNF can alleviate JC in both IBD patients with arthralgia and IBD patients with arthritis (16). Ustekinumab, targeting IL-12 and IL-23, also alleviates arthralgia in IBD patients, but is not effective for the treatment of axial SpA (axSpA) (17, 18). Conversely, some IBD patients develop *de novo* arthralgia under ustekinumab treatment (17). These findings suggest that the biological mechanisms underlying the symptoms of IBD and SpA may differ per patient, raising the question of whether treatment strategies should be tailored accordingly.

To explore whether joint complaints in IBD are associated with systemic immune features overlapping with SpA, we analyzed serum samples from two established cohorts using a proximity extension assay targeting 92 inflammation-related proteins. Serum does not fully capture tissue-localized immune processes in the gut or joints, but it may reflect systemic inflammatory pathways relevant to both disease domains. The selected panel includes proteins linked to key immune axes implicated in IBD and SpA, including IL-6, IL-12B, IL-17A, TNF-related proteins, interferon-inducible chemokines such as CXCL9, CXCL10 and CXCL11, innate inflammatory mediators such as OSM and EN-RAGE, and remodeling-associated proteins such as HGF, VEGFA and TGF-α. We compared IBD patients with and without joint complaints, IBD patients with joint complaints with and without SpA, and chronic back pain patients with and without proven axSpA. By integrating differential protein abundance, unsupervised clustering, functional protein signatures, and predictive modeling, we aimed to determine whether circulating immune profiles align more strongly with formal SpA diagnosis or with the presence of joint symptoms.

## 2 Methods

### 2.1 Ethics statement

The study protocols were approved by the medical ethical committee of the Leiden University Medical Center, and patients signed a written informed consent before study enrolment.

### 2.2 Cohorts

For this study, the serum of patients included in two Dutch cohorts were analyzed: (i) the JOINT cohort described by van Erp et al. (19), and (ii) the LUMC SPACE cohort described by Marques et al. (20). The prospective JOINT cohort includes 255 IBD patients with (n = 155) and without (n = 100) self-reported joint complaints (JC). JC are defined as chronic back pain (CBP) for ≥ 3 months and/or peripheral joint complaints currently or during the previous year. All IBD patients underwent a rheumatological examination by a well-trained clinical researcher. Based on this examination, patients were classified according to several clinical SpA criteria (ASAS, Amor, and ESSG). Patients with evident joint swelling and signs of inflammation (physical examination and/or on radiographs) were referred to the rheumatologist (n = 52), who diagnosed 15 patients with axial and/or peripheral SpA. Fecal calprotectin values were available for a limited number of patients and, therefore, were not considered in this study. Medication use was determined at time of blood draw. Of the cohort, 27% of patients with and 20% of patients without JC did not use any medication for their IBD. The other patients used various medication.

The LUMC SPACE cohort consists of 169 patients under the age of 45 presenting with chronic back pain (CBP) of recent onset (≥3 months, ≤2 years) and unknown origin at the rheumatology outpatient clinic. These patients were followed for 2 years, after which a rheumatologist reported a clinical diagnosis of axSpA or non-axSpA with a level of confidence (LoC) varying from 0 (not confident at all) to 10 (very confident). In the current analysis, only diagnoses with LoC ≥7, or with LoC <7 at 2 years plus a consistent diagnosis in the two last visits, or for non-axSpA with LoC <7 plus an alternative reported diagnosis were included (n = 164). Patients with the diagnosis IBD were excluded (n = 9). Only serum samples from baseline were analyzed. Patients did not use any disease-modifying antirheumatic drugs at baseline.

### 2.3 Proximity extension assay

Serum concentrations of 92 inflammation-related proteins were analyzed using PEA technology using the ProSeek Multiplex Inflammation panel from Olink Proteomics^®^ (see **Supplementary Table 1** for a list of names, abbreviations, UniProt IDs, limits of detection (LODs), and detection rates). Oligonucleotide-labelled antibodies (probes) were incubated with the samples, which enabled pair-wise binding of the probes to the target proteins. When two probes of the same type come in close proximity, they hybridize, allowing DNA polymerase extension. The resulting PCR target sequence is subsequently detected and quantified using real-time microfluidic quantitative polymerase chain reaction (qPCR). The relative normalized protein expression (NPX) values (on an arbitrary log2 scale) of individual proteins can be compared across samples; however NPX values for different proteins cannot be compared directly.

To ensure assay performance and sample quality, internal controls were added to samples. Samples that deviated >0.3 NPX from the median of the internal controls failed quality control and were excluded (n = 4 in the SPACE cohort; n = 14 in the JOINT cohort). In the JOINT cohort, 18 proteins were below the LOD in >20% of serum samples, and were excluded. These 18 proteins also had low detection rates in the SPACE cohort, and were therefore also excluded in this cohort. In addition, TNF-α was excluded from the JOINT cohort, because some patients in this cohort used anti-TNF medication (infliximab or adalimumab; 32% in patients with JC and 32% in patients without JC). The antibodies from this therapy can form complexes with TNF-α which may be detected by the Olink assay, possibly leading to skewed measurements (21). For the remaining proteins, NPX values below the LOD were treated as missing values. Single imputation of the missing values by using half the LOD value did not alter the results unless stated otherwise.

### 2.4 Statistical analysis

Demographic and clinical characteristics of the cohorts are presented as proportions *n* with corresponding percentages (%) or medians with interquartile range. Normality was assessed by visual inspection of histograms and normal probability (Q–Q) plots. Differences between groups were tested using Mann–Whitney U-tests, Pearson’s chi-square tests, or Fisher’s exact tests, depending on the type of variable and group sizes.

False discovery rates (FDR) <0.05, adjusted for multiple comparisons using the Benjamini-Hochberg procedure, were considered significant. For group comparisons, multivariate linear regression models were used. In each case, one model was performed per grouping variable versus each serum protein, correcting for sex, age, elevated C-reactive protein (CRP) (≥5 mg/L), smoking status (yes/no), medication use, and IBD type. Hierarchical clustering of patients was performed using Euclidean distance and complete linkage. Clustering was based on the top 10 proteins with the highest median absolute deviation (MAD). Additionally, to characterize functional inflammatory modules, four protein signatures were defined: cytotoxic, Th17/neutrophil, innate, and remodeling. The cytotoxic signature comprised CD8A, CXCL9, CXCL10, CXCL11, IFN-gamma, IL-12B, IL18, TNFRSF9, and TRAIL. The Th17/neutrophil signature consisted of CCL20, CXCL1, CXCL5, CXCL6, IL6, IL8, IL-12B, and IL-17A. Proteins included in the innate signature were CCL3, CCL4, CX3CL1, CSF-1, EN-RAGE, IL6, IL18, MCP-1, MCP-2, MCP-3, MCP-4, OSM, and uPA. The remodeling signature included HGF, MMP-1 MMP-10, TGF-alpha, and VEGFA. For each sample, a signature score was calculated as the mean of z-scores of the proteins in the signature. Internal consistency of each signature was assessed using Cronbach’s alpha.

To evaluate if serum immune protein profiles can distinguish patient groups better than chance, predictive modeling was performed using Least Absolute Shrinkage and Selection Operator (LASSO) regression. Ten-fold cross-validations was applied, and the model corresponding to the λ-value within one standard error of the minimum cross-validated error (λ_1_se) was selected. This is a slightly more conservative regularization parameter than the one minimizing cross-validation error, to reduce the risk of overfitting. As LASSO regression does not accommodate missing values, these were imputed using half the LOD value of each respective protein. The area under the curve (AUC) of the Receiver Operating Characteristic (ROC) curve was used as a metric to evaluate model performance. Predictive modeling was not performed on comparisons with the group of IBD patients with SpA because of the small sample size of this group (n = 15), which would yield unreliable model estimates regardless of the cross-validation strategy employed. Correlations between serum proteins and disease parameters were assessed using Spearman’s correlation. In all analyses, IBD patients (n = 9) were excluded from the SPACE cohort. All analyses were performed using R version 4.3.3.

Protein-protein interaction (PPI) networks were constructed using the multi-protein model from the Search Tool for the Retrieval of Interacting Genes (STRING) online platform (version 12.0) with direct (physical) interactions defined by a confidence score of ≥0.4 (22).

## 3 Results

**Figure 1:**
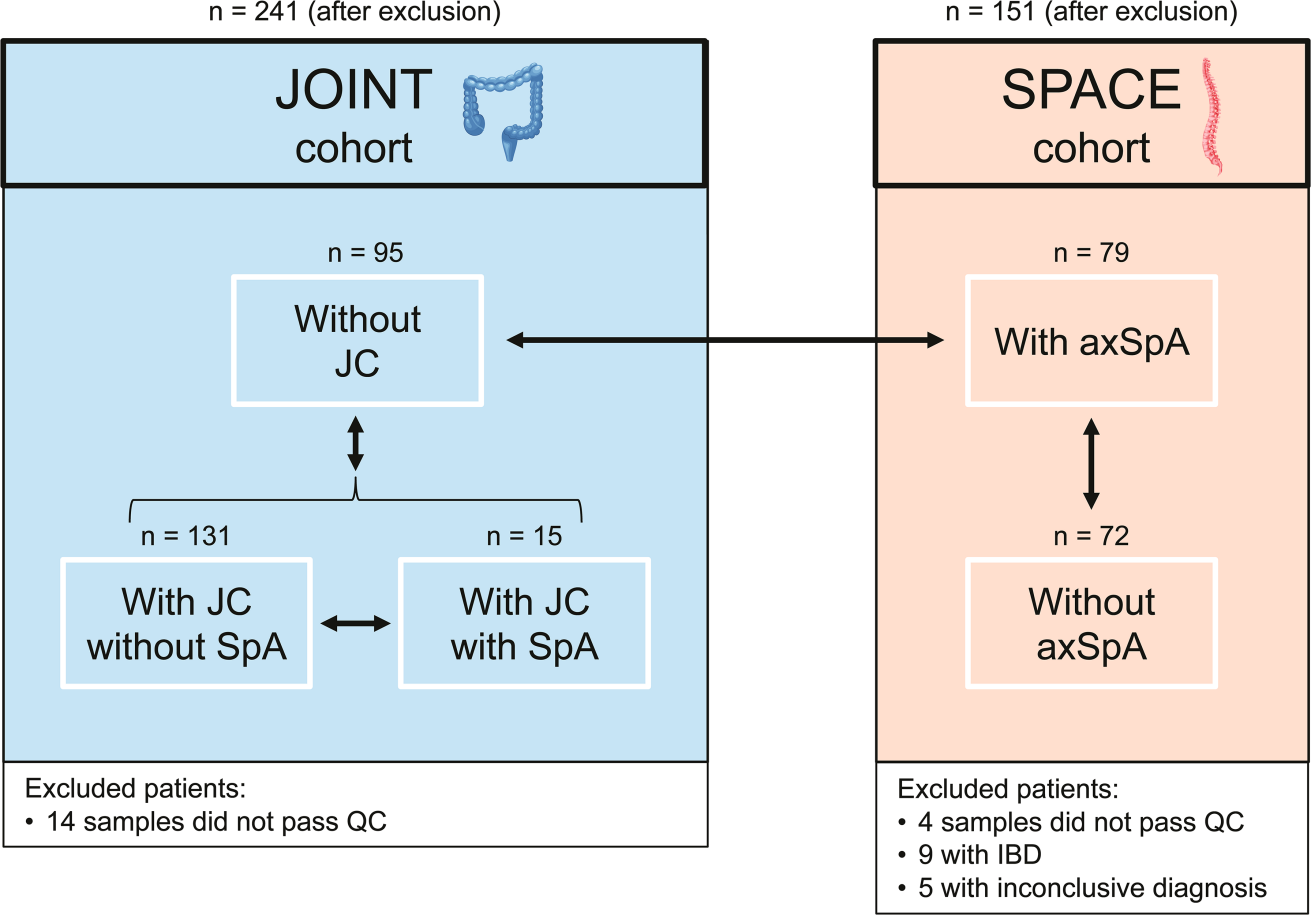
Graphical overview of the cohorts and group comparisons (comparisons indicated with arrows). In the JOINT cohort, SpA diagnosis was determined at the moment the serum sample was taken, while in the SPACE cohort, axSpA diagnosis was determined two years after serum sample collection. axSpA, axial spondyloarthritis; IBD, inflammatory bowel disease; JC, joint complaints; QC, quality control; SpA, spondyloarthritis.

Within and between the JOINT and SPACE cohort, several groups of patients were compared, as illustrated in **Figure 1**. A summary of the demographics and clinical characteristics of these patient groups is presented in **Table 1**. Among IBD patients, no significant differences in serum immune protein profiles were observed between those with UC and CD.

**Table 1:** Demographic and clinical characteristics of the JOINT and SPACE cohorts.

Parameter	JOINT cohort			SPACE cohort		
	Without JC n = 95	With JC n = 146	p-value	Without axSpA n = 72	With axSpA n = 79	p-value
Female gender	47 (49%)	101 (69%)	< 0.01	57 (79%)	35(44%)	< 0.001
Age (years)	42 (33.5-53)	42 (33.25-54)	0.826	30.5 (22–38)	26(23-36)	0.338
Current smoking	13 (14%)	45 (31%)	< 0.01	18 (25%)^a^	21(27%)^b^	1
CRP (mg/L)	3 (3-4)	3 (3-5.75)	0.169	3 (3-7.15)	3(3-9)	0.124
HLA-B27+	0 (0%)	6 (4%)	0.115	12 (17%)	63(80%)	< 0.001
ESR	9 (2-14)^c^	9 (6-19.75)^d^	< 0.05			
IBD type						
Crohn’s disease	61 (64%)	115 (79%)	0.019	–	–	
Ulcerative colitis	34 (36%)	31 (21%)		–	–	
Medication use						
5-ASA (mesa, sulfa)	33 (35%)	32 (22%)	0.041	–	–	
Steroids	11 (12%)	16 (11%)	1	–	–	
Immunosuppressive	35 (37%)	51 (35%)	0.869	–	–	
drugs (Aza/6MP/MTX)				–	–	
Anti-TNF	30 (32%)	46 (32%)	1			
No medication	19 (20%)	40 (27%)	0.2494			
Disease activity score						
HBI/SCCAI	19 (20%)	76 (52%)	< 0.001	–	–	
(active disease ≥5)						
ASDAS	–	–		2.68 (2.10-3.39)^d^	2.53 (1.91-3.11)^e^	0.359
BASMI	–	–		2.2 (1.8-3.0)^f^	1.6 (1.2-2.4)^g^	0.079
SpA						
Meets ASAS, Amor, and/or ESSG criteria	58 (40%)		–	–	
Meets ASAS criteria	–	8 (5%)		–	–	
Meets Amor criteria	–	26 (18%)		–	–	
Meets ESSG criteria	–	45 (31%)		–	–	
Diagnosis according to rheumatologist	–	15 (10%)		–	–	

Data are presented as proportions n with corresponding percentages (%) or median (interquartile range) in case of continuous variables.

JC, joint complaints; axSpA, axial spondyloarthritis; ESR, erythrocyte sedimentation rate; 5-ASA (mesa, sulfa), 5-aminosalicylates (mesalazine, sulfasalazine); AZA, azathioprine; 6MP, 6-mercaptopurine; MTX, methotrexate; TNF, tumour necrosis factor; HBI, Harvey-Bradshaw Index; SCCAI, Simple Clinical Colitis Activity Index; ASDAS, Axial Spondyloarthritis Disease Activity Score; BASMI, Bath Ankylosing Spondylitis Metrology Index; ASAS, Assessment of SpondyloArthritis international Society; ESSG, European Spondyloarthropathy Study Group.

^a^ Four missing data points.

^b^ Three missing data points.

^c^ Two missing data points.

^d^ Two missing data points.

^e^ Six missing data points.

^f^ Three missing data points.

^g^ Two missing data points.

### 3.1 IBD patients without joint complaints and axSpA patients have distinct serum immune profiles

**Figure 2:**
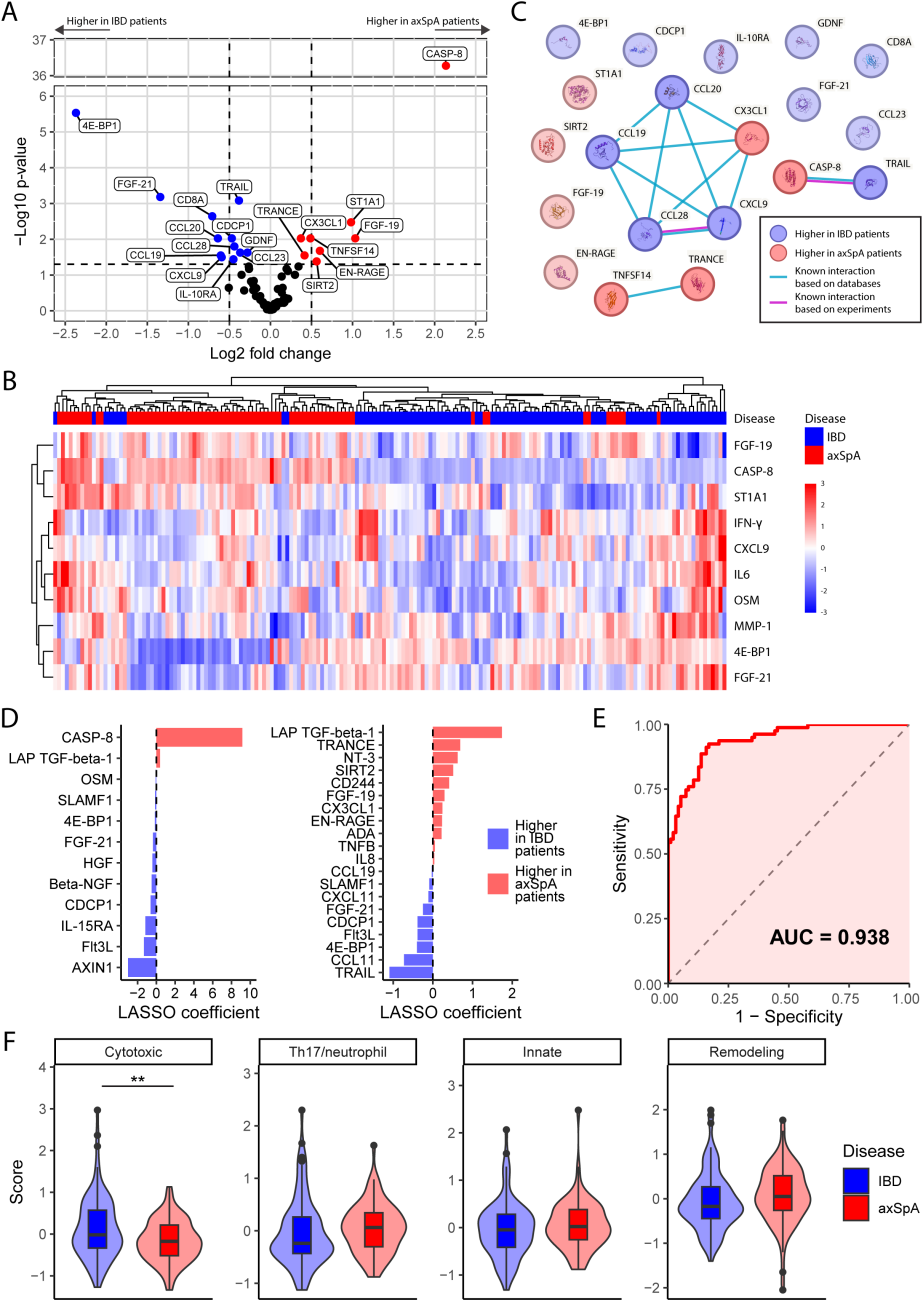
Comparison of IBD patients without joint complaints (n = 95) to axSpA patients (n = 79). **(A)** Volcano plot showing 20 proteins are differentially abundant between IBD and axSpA patients. Horizontal dotted line indicates threshold for significance (FDR <0.05) after correction for multiple testing based on the Benjamini-Hochberg procedure. **(B)** Heatmap of 10 proteins with highest MAD with hierarchical clustering (Euclidean distance, complete linkage) shows groups of IBD patients and groups of axSpA patients clustering together. **(C)** PPI network of differentially abundant proteins between axSpA and IBD patients, established using the STRING database. **(D)** LASSO coefficients of the selected proteins at λ_1_se, using either all proteins as input (left) or excluding CASP-8 (right). **(E)** ROC curve of the LASSO model at λ_1_se, with CASP-8 excluded as input, and the corresponding AUC indicated. **(F)** Violin and boxplots of signature scores. Boxplots indicate median (center line), interquartile range (box), and whiskers extending to 1.5x the interquartile range (IQR). AUC, area under the curve; axSpA, axial spondyloarthritis; FDR, false discovery rate; IBD, inflammatory bowel disease; LASSO, Least Absolute Shrinkage and Selection Operator; MAD, median absolute deviation; PPI, protein-protein interaction; ROC, receiver operating characteristic; STRING, Search Tool for the Retrieval of Interacting Genes. **p<0.01.

First, we investigated whether the serum immune profile of patients with different inflammatory diseases, IBD and axSpA, are different (**Figure 1**). The serum concentrations of 20 proteins were significantly different between IBD patients without joint complaints (JC) (n = 95) and axSpA patients (n = 79): 12 proteins were increased in IBD patients, and 8 proteins were increased in axSpA patients (**Figure 2A**) (functional description provided in **Supplementary Table 2**). Notably, 4E-BP1 and caspase-8 had relatively high values in IBD and axSpA patients, respectively. Unsupervised hierarchical clustering based on the 10 proteins with the highest median absolute deviation (MAD) revealed that patients predominantly clustered according to their diagnosis (**Figure 2B**), indicating that IBD patients without JC and axSpA patients have distinct serum immune profiles. Single imputation of missing values using half the LOD value marginally changed the results: the difference in IL-10RA protein concentration was not significant anymore, and the patients clustering was slightly different (**Supplementary Figure 1**). Unsupervised hierarchical clustering based on all proteins is shown in **Supplementary Figure 2**.

To further investigate the differentially abundant proteins, we performed STRING database analysis to construct a protein-protein interaction (PPI) network (**Figure 2C**). The network revealed only one known interaction between proteins increased in axSpA patients, while four proteins increased in IBD patients showed interactions. Notably, many proteins fall outside the PPI network, highlighting the complexity of these diseases, as multiple pathways appear to be affected.

To assess whether serum immune profiles can distinguish patient groups, we performed predictive modeling using LASSO regression. The selected model identified 12 proteins as discriminating features, with CASP-8 showing the largest coefficient, consistent with the differential expression analysis (**Figure 2D**). Many of the other selected proteins were not significant in the differential abundance analysis, suggesting that predictive modeling captures complementary information. ROC curve analysis revealed an AUC of 1.00, indicating that the model discriminated the serum immune profiles of IBD patients and axSpA patients with high accuracy driven largely by CASP-8. After excluding CASP-8, the model maintained high discriminatory capacity, with the ROC curve showing an AUC of 0.938 (**Figure 2E**). These results suggest IBD patients without JC and axSpA patients can be distinguished based on their serum immune profiles.

Comparison of functional inflammatory signatures revealed that IBD patients without JC had significantly higher cytotoxic signature scores compared to axSpA patients, while Th17/neutrophil, innate, and remodeling signature scores did not differ between groups (**Figure 2F**). To evaluate whether the predefined protein signatures represent coherent biological modules, we assessed internal consistency using pairwise protein-protein correlations and Cronbach’s alpha (**Supplementary Figure 3**). Cronbach’s alpha values ranged from 0.61 to 0.83, indicating moderate to good internal consistency.

### 3.2 Presence of SpA minimally impacts serum immune profile in patients with joint complaints

**Figure 3:**
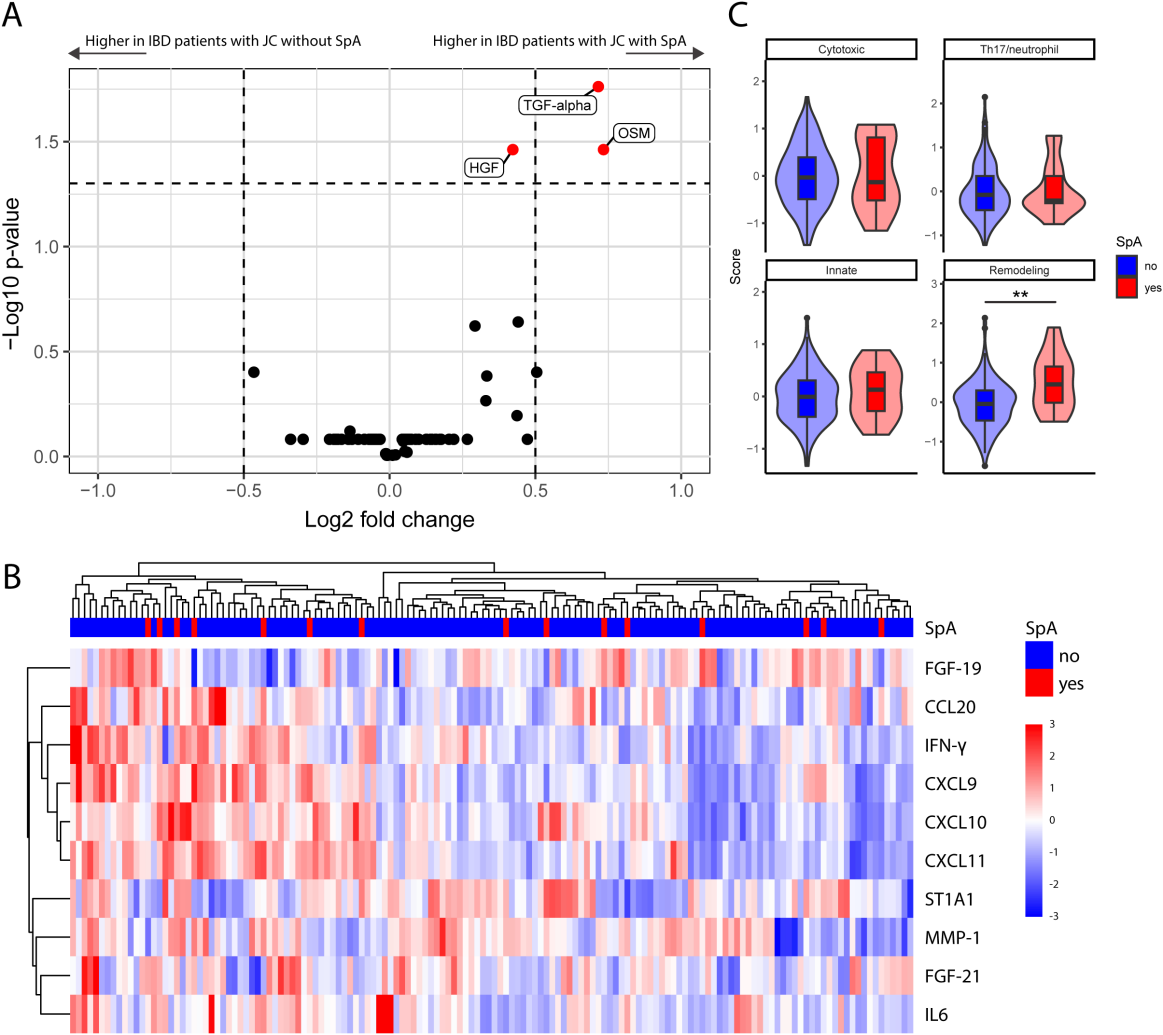
Comparison of IBD patients with joint complaints with (n = 15) versus without (n = 131) SpA. **(A)** Volcano plot showing 3 proteins are significantly increased in serum of IBD patient with SpA compared to IBD patients with JC but without SpA. Horizontal dotted line indicates threshold for significance (FDR <0.05) after correction for multiple testing based on the Benjamini-Hochberg procedure. **(B)** Heatmap of 10 proteins with highest MAD with hierarchical clustering (Euclidean distance, complete linkage) shows patients with SpA diagnosis do not cluster together. **(C)** Violin and boxplots of signature scores. Boxplots indicate median (center line), IQR (box), and whiskers extending to 1.5x the IQR. FDR, false discovery rate; IBD, inflammatory bowel disease; IQR, interquartile range; JC, joint complaints; MAD, median absolute deviation; SpA, spondyloarthritis. **p<0.01.

We further investigated whether serum immune profiles of IBD patients with JC differ between those with (n = 15) and without (n = 131) SpA. Only three proteins, TGF-α, OSM, and HGF, had significantly higher concentrations in the serum of IBD patients with SpA (**Figure 3A**). Unsupervised hierarchical clustering of IBD patients with JC based on the 10 proteins with the highest MAD revealed that those patients with a SpA diagnosis did not cluster together (**Figure 3B**). This suggests that whether or not an IBD patient with JC has a SpA diagnosis minimally impacts their serum immune profile. A difference was observed in remodeling signature, which was significantly higher in IBD patients with SpA compared to IBD patients with JC without SpA (**Figure 3C**).

**Figure 4:**
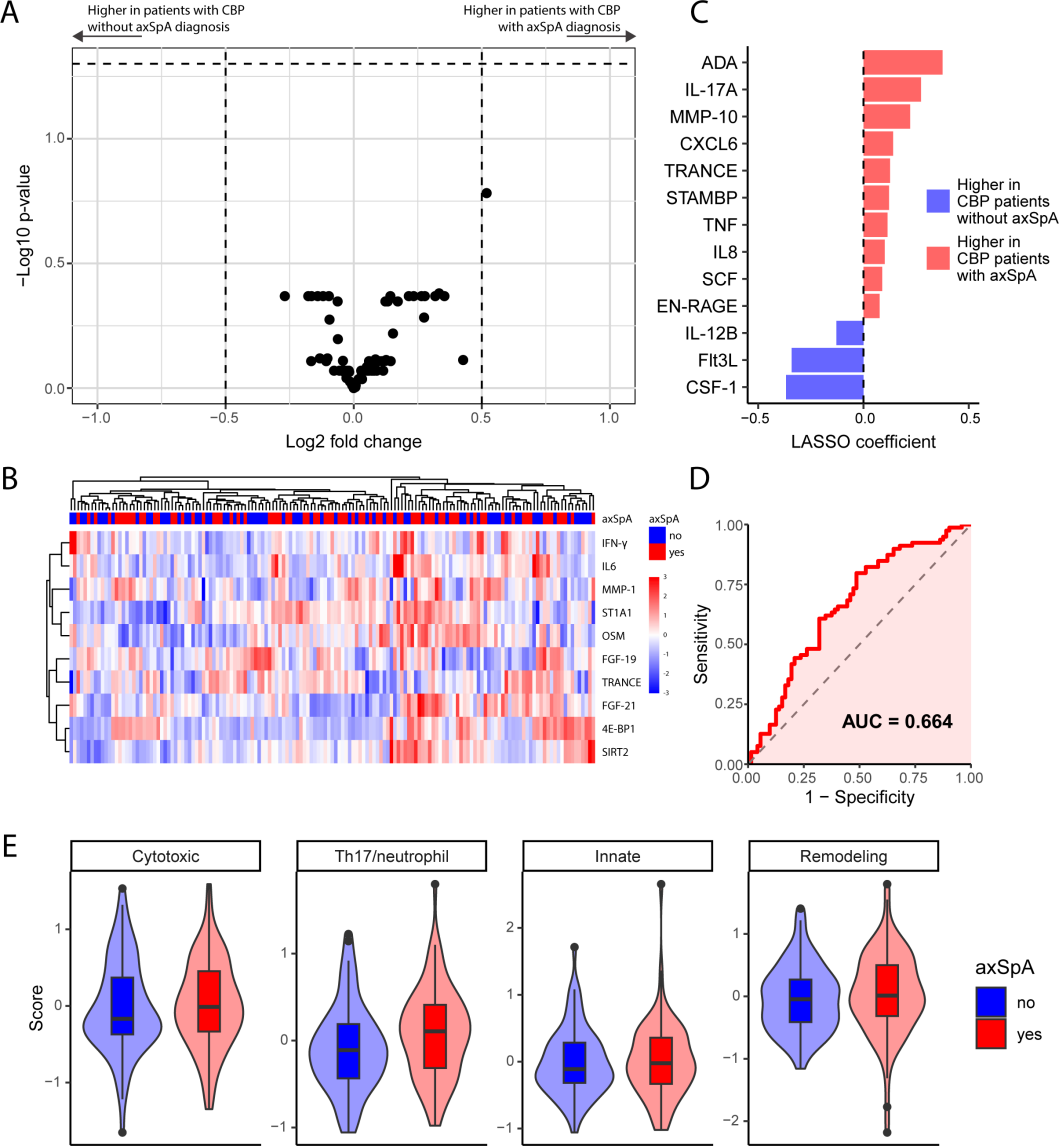
Comparison of patients with CBP with (n = 79) versus without (n = 72) axSpA diagnosis. **(A)** Volcano plot showing no differentially abundant proteins in serum of patients with CBP with compared to without axSpA diagnosis. Horizontal dotted line indicates threshold for significance (FDR <0.05) after correction for multiple testing based on the Benjamini-Hochberg procedure. **(B)** Heatmap of 10 proteins with highest MAD with hierarchical clustering (Euclidean distance, complete linkage) shows patients with axSpA diagnosis do not cluster together. **(C)** LASSO coefficients of the selected proteins at λ_1_se. **(D)** ROC curve of the LASSO model at λ_1_se, with AUC indicated. **(E)** Violin and boxplots of signature scores. Boxplots indicate median (center line), IQR (box), and whiskers extending to 1.5x the IQR. AUC, area under the curve; axSpA, axial spondyloarthritis; CBP, chronic back pain; FDR, false discovery rate; IQR, interquartile range; LASSO, Least Absolute Shrinkage and Selection Operator; MAD, median absolute deviation; ROC, receiver operating characteristic.

In the SPACE cohort, we also investigated the difference in serum immune profile between patients with JC (also referred to as CBP) with and without subsequent (ax)SpA diagnosis in the SPACE cohort (non-IBD patients). No proteins were significantly different between these two groups (**Figure 4A**). Again, unsupervised hierarchical clustering of the patients based on the 10 proteins with the highest MAD showed no clustering of the patients based on their axSpA diagnosis (**Figure 4B**). Additionally, predictive modeling using LASSO regression selected 13 proteins, but the model showed poor discriminatory capacity with an AUC of 0.664 (**Figures 4C, D**). Comparison of signature scores showed no differences between groups (**Figure 4E**), suggesting an axSpA diagnosis was not associated with major changes in serum immune profile.

### 3.3 Joint complaints in IBD patients associated with differences in serum immune profile

**Figure 5:**
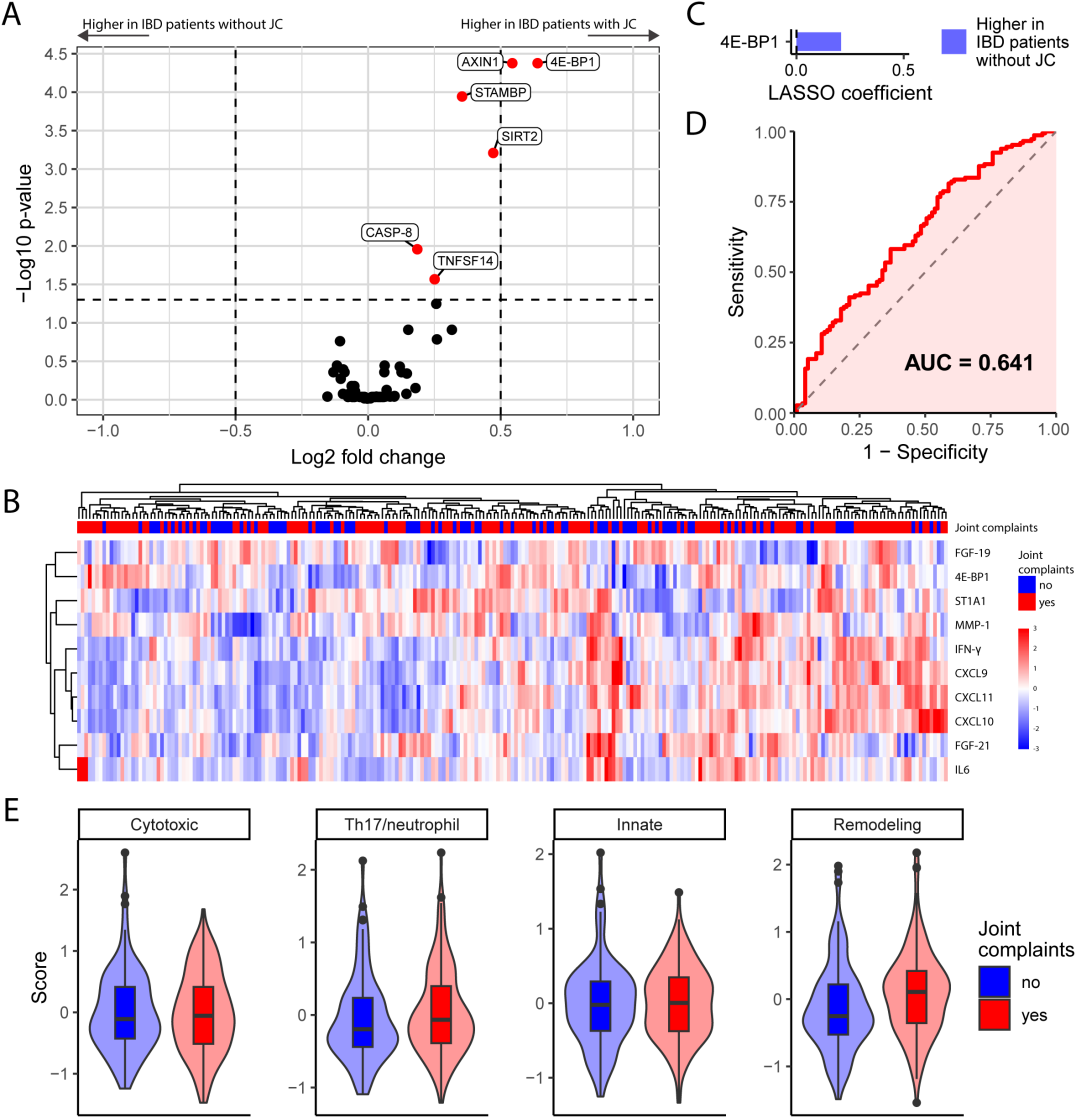
Comparison of IBD patients with versus without joint complaints. **(A)** Volcano plot showing comparisons of IBD patients without JC (n = 95) to IBD patients with JC (n = 146). Horizontal dotted line indicates threshold for significance (FDR <0.05) after correction for multiple testing based on the Benjamini-Hochberg procedure. **(B)** Heatmap of 10 proteins with highest MAD with hierarchical clustering (Euclidean distance, complete linkage) shows patients with SpA diagnosis do not cluster together. **(C)** LASSO coefficients of the selected proteins at λ_1_se. **(D)** ROC curve of the LASSO model at λ_1_se, with AUC indicated. **(E)** Violin and boxplots of signature scores. Boxplots indicate median (center line), IQR (box), and whiskers extending to 1.5x the IQR. AUC, area under the curve; FDR, false discovery rate; IBD, inflammatory bowel disease; JC, joint complaints; IQR, interquartile range; LASSO, Least Absolute Shrinkage and Selection Operator; MAD, median absolute deviation; ROC, receiver operating characteristic; SpA, spondyloarthritis.

We further investigated whether the serum immune profiles of IBD patients with and without JC differ within the JOINT cohort. Six proteins were significantly increased in IBD patients with JC: 4E-BP1, AXIN1, STAMBP, SIRT2, CASP-8, and TNFSF14 (**Figure 5A**). Three of these proteins, SIRT2, CASP-8, and TNFSF14, were also increased in axSpA patients compared to IBD patients without JC (**Table 2**). Interestingly, one protein that was increased in IBD patients with JC, 4E-BP1, showed a higher concentration in the serum of IBD patients compared to axSpA patients. Unsupervised hierarchical clustering of the patients from the JOINT cohort, based on the 10 proteins with the highest MAD, showed that patients with JC did not cluster together (**Figure 5B**). Predictive modeling using LASSO regression retained only 4E-BP1 as a discriminating feature (**Figure 5C**). ROC curve analysis showed an AUC of 0.641, indicating poor discriminatory capacity (**Figure 5D**). Furthermore, the remodeling signature score was higher in IBD patients with JC compared to those without JC, although this difference was not significant (**Figure 5E**). These results suggest that while IBD patients with and without JC have slightly different serum immune profiles, the presence of JC is not a major determinant of serum immune profiles among IBD patients. IBD patients with JC had higher disease activity (HBI or SCCAI) than those without JC (**Table 1**), however no proteins were differentially abundant between patients with and without active disease (**Supplementary Figure 4A**).

**Table 2:** Overview of differentially abundant proteins in various group comparisons.

Protein	Increased in IBD patients without JC (n = 95) vs axSpA patients (n = 79)	Increased in axSpA patients (n = 79) vs IBD patients without JC (n = 95)	Increased in IBD patients with JC with (n = 15) vs without SpA (n = 131)	Increased in CBP patients with (n = 79) vs without (n = 72) axSpA	Increased in IBD patients with (n = 146) vs without (n = 95) JC	Increased in IBD patients who meet SpA criteria (n = 58) vs without JC (n = 95)	Increased in IBD patients with SpA diagnosis (n = 15) vs without JC (n = 95)
CASP-8	–	X	–	–	X	X	X
SIRT2	–	X	–	–	X	X	X
AXIN1	–	–	–	–	X	X	X
STAMBP	–	–	–	–	X	X	X
TNFSF14	–	X	–	–	X	–	X
4E-BP1	X	–	–	–	X	X	–
HGF	–	–	X	–	–	–	X
OSM	–	–	X	–	–	–	X
TGF-α	–	–	X	–	–	–	X
CX3CL1	–	X	–	–	–	–	–
EN-RAGE	–	X	–	–	–	–	–
FGF-19	–	X	–	–	–	–	–
ST1A1	–	X	–	–	–	–	–
TRANCE	–	X	–	–	–	–	–
CCL19	X	–	–	–	–	–	–
CCL20	X	–	–	–	–	–	–
CCL23	X	–	–	–	–	–	–
CCL28	X	–	–	–	–	–	–
CD8A	X	–	–	–	–	–	–
CDCP1	X	–	–	–	–	–	–
CXCL9	X	–	–	–	–	–	–
GDNF	X	–	–	–	–	–	–
FGF-21	X	–	–	–	–	–	–
IL-10RA	X	–	–	–	–	–	–
TRAIL	X	–	–	–	–	–	–

We then compared IBD patients without JC to a subgroup of IBD patients with JC who also met at least one of the clinical SpA classification criteria, including ASAS, Amor, and ESSG criteria. The same proteins that were increased in the broader group of IBD patients with JC (4E-BP1, AXIN1, STAMBP, SIRT2, and CASP-8) were also increased in this subgroup, with the exception of TNFSF14 (**Supplementary Figure 4B**).

Further comparison of IBD patients without JC to a more specific subgroup of IBD patients with JC and a SpA diagnosis revealed an increase of the same proteins (including TNFSF14), except for 4E-BP1 (**Supplementary Figure 4C**). Additionally, TGF-α, HGF, and OSM were increased in IBD patients with JC and an SpA diagnosis compared to those without JC. These proteins were also observed in **Figure 3A**, which compared IBD patients with JC with and without SpA. The fold changes in all these comparisons were relatively small, with a maximum fold change of 0.80 on the Log2 scale, equivalent to a 1.74-fold change on the linear scale.

Overall, although IBD patients with JC do not cluster together in unsupervised hierarchical clustering, patients with and without JC exhibit different serum immune profiles. The difference in immune profile is also evident in more specific subgroups of patients with JC.

### 3.4 Correlation between disease parameters and serum protein concentrations

Finally, we investigated the correlation between disease parameters and serum protein concentrations. In the JOINT cohort, erythrocyte sedimentation rate (ESR) correlated significantly with some of the proteins, while no significant correlations were observed with HLA-B27 positivity, C-reactive protein (CRP), or disease activity (**Supplementary Figure 5**). Disease activity was defined by dichotomizing the HBI and SCCAI scores with a cut-off value of ≥5. In the SPACE cohort, BASMI, CRP, and ASDAS correlated significantly with some proteins, whereas HLA-B27 positivity did not (**Supplementary Figure 5**). The strongest correlation was between CRP and IL6. However, all correlation coefficients were below |0.4|, indicating weak correlations.

## 4 Discussion

This study demonstrates that IBD patients without joint complaints (JC) and axSpA patients without IBD have distinct serum immune protein profiles. Among IBD patients with self-reported JC and among patients with self-reported chronic back pain, the serum immune profile is generally uniform, regardless of SpA diagnosis. Notably, however, IBD patients with and without JC show differences in serum immune profile. These findings suggest that joint complaints in IBD may capture biologically relevant immune variation that extends beyond current SpA classification. The key finding is that joint complaints in IBD are associated with a modest serum immune shift, partly overlapping with axSpA-associated proteins, while formal SpA diagnosis adds limited additional serum separation among patients who already have joint symptoms.

In the comparison between IBD patients without JC and axSpA patients, the proteins 4E-BP1 and CASP-8 showed the largest differences in abundance, with 4E-BP1 being higher in IBD patients, and CASP-8 being higher in axSpA patients. CASP-8 can induce apoptosis and seems to have both protective and pathogenic effects in IBD, while its role in axSpA is unclear (23). Interestingly, other studies found that CASP-8 is increased in IBD patients compared to healthy controls (24–26), but it is not elevated in axSpA patients compared to healthy controls (27). 4E-BP1 is a repressor of translation initiation that acts downstream of mTORC1, which like CASP-8 seems to have both protective and pathogenic effects in IBD (28, 29). The exact role of 4E-BP1 in axSpA is unknown, but mTORC1 is activated in the joints of SpA patients, and inhibition of mTORC1 in a rat SpA model reduced SpA development and severity (30). Although most prior studies examining 4E-BP1 serum concentrations reported no differences between IBD or axSpA patients and controls, one study found increased levels in IBD patients compared with controls (24–27). A potential limitation of the current study is that most IBD patients, but no axSpA patients, were on anti-inflammatory medication, which may have influenced serum protein levels.

A substantial portion of the proteins that was increased in the serum of IBD patients compared to axSpA patients are chemotactic molecules that attract T-cells. These proteins include CCL19, CCL20, CCL28, and CXCL9, which form an interaction network as displayed in **Figure 2C**. Given the important role of T-cells in IBD pathogenesis, it is expected that IBD patients have elevated levels of T-cell chemoattractants compared to healthy controls. Additionally, our data show that IBD patients have increased cytotoxic signature scores. However, this study compared IBD patients to axSpA patients, and T-cells also play a role in the pathogenesis of axSpA. It could be that these chemoattractants for T-cells are elevated in both IBD and in axSpA patients (compared to healthy controls), but that they are more elevated in IBD patients. Previous studies demonstrated that CCL19, CCL20, CCL28, and CXCL9 are indeed elevated in IBD patients compared to symptomatic or healthy controls (24, 26). Conversely, other studies found no difference in plasma levels of these T-cell attractants between IBD patients and healthy controls, but did observe elevated levels of CXCL11, another T-cell attractant, in IBD patients (25). No differences in T-cell chemoattractants plasma levels were found in axSpA patients compared to healthy controls (27). So, at least some T-cell chemoattractants are elevated in the serum of IBD patients, and although T-cells are involved in the pathogenesis of axSpA, these chemoattractants are not elevated in the serum of axSpA patients.

Although IL-17A contributes to SpA pathogenesis and exerts a protective role in IBD, no significant difference in IL-17A concentration was observed between these groups. This finding may be attributed to the measurement of IL-17A in serum rather than at disease-affected sites, such as the spine or gut. Previous studies have reported elevated IL-17A serum concentrations in IBD patients compared to healthy controls (24–26), while studies with axSpA patients show inconsistent results (27, 31). The protective role of IL-17A in IBD may be mediated by various mechanisms, including the enhancement of mucosal barrier integrity and mucosal defense against infection through neutrophil recruitment and stimulation of antimicrobial peptide production (32). Furthermore, an anti-IL-17 antibody enhanced disease severity in a DSS colitis mouse model (33). Clinical trials with anti-IL-17 antibodies in CD patients showed no efficacy or even worsening of CD symptoms (34, 35). In contrast, three different IL-17 inhibitors have been approved by the U.S. Food and Drug Administration and the European Medicines Agency for treatment of SpA (36). IL-17 contributes to SpA pathogenesis by inducing pro-inflammatory cytokines and chemokines and stimulating osteoclast-mediated bone resorption through upregulation of TRANCE, which stimulates osteoclast differentiation (36). Given its opposing roles in IBD and SpA, IL-17 is unlikely to represent a primary shared mechanism underlying the gut-joint association, pointing towards other mediators as more plausible drivers of this relationship.

Except for IL-17A, the serum concentrations of numerous inflammatory proteins differ between IBD and axSpA patients, suggesting the type of inflammatory disease affects the serum immune profile. In contrast, among IBD patients with self-reported JC and among patients with self-reported CBP, the serum immune profiles are largely similar, irrespective of SpA diagnosis. In line with this, predictive modeling using LASSO regression showed poor discriminatory performance between CBP patients with and without axSpA diagnosis. Between IBD patients with JC with and without SpA, only three proteins (OSM, HGF, and TGF-α) were differentially abundant. HGF and TGF-α are part of the remodeling signature, which also scored significantly higher in IBD patients with SpA. Interestingly, the three differentially abundant proteins correspond to three of the four proteins Jongsma et al. (37) found to be associated with a severe disease course in therapy naïve moderate to severe pediatric CD patients. In the current study, 14 out of 15 IBD patients with SpA were diagnosed with CD, which may introduce bias into the analysis. However, comparative analysis between CD and UC patients did not reveal any differentially abundant proteins. It is important to note that the analyzed samples from the SPACE cohort were collected two years prior to the eventual determination of SpA diagnosis, and potential changes in the serum immune profile during this period remain unknown. Furthermore, a direct comparison between patients with JC or CBP and healthy controls is lacking and warrants further investigation. In addition, the lack of observed differences in serum immune profiles does not preclude the presence of other differences beyond the scope of the assay used here. Despite these limitations, the current findings provide preliminary evidence that the presence of SpA in patients with IBD and JC or in patients with CBP may not significantly alter the systemic immune protein profile, as reflected in serum protein concentrations.

Moreover, the serum immune profiles of IBD patients with and without JC do differ somewhat, with a partial overlap in increased proteins between IBD patients with JC and axSpA patients. Nevertheless, these differences remain small, with a maximum fold change of 1.56. Comparison of IBD patients without JC to a subgroup of IBD patients with JC and a SpA diagnosis showed an increase of mostly the same proteins as comparison to the full group of IBD patients with JC. Other studies also found differences in the blood between IBD patients and patients with both IBD and SpA: Lefferts et al. (38) found that patients with Crohn’s disease and axSpA (CD-axSpA) have elevated levels of granzyme B+ T-cells compared to patients with only CD. In addition, they showed that CD-axSpA patients have elevated plasma concentrations of IL-6. They did not, however, specifically investigate the immune profile of CD patients with JC but without SpA. The biological distinction between arthralgia and SpA remains relatively unexplored. Our data suggest that, at least at the level of circulating inflammatory proteins, formal SpA diagnosis does not define a strongly distinct subgroup among IBD patients who already report joint complaints. Instead, joint complaints themselves may mark a broader biologically relevant subgroup within IBD.

Current IBD treatment guidelines distinguish between different forms of IBD-associated arthropathy, and in clinical practice treatment decisions for joint symptoms often depend on whether a patient receives a formal rheumatological diagnosis (39, 40). Our findings raise the possibility that this diagnostic boundary may not fully capture the underlying immune biology of joint symptoms in IBD. In particular, IBD patients with JC but without formal SpA may still show serum immune changes that partly overlap with axSpA-associated proteins. It supports the need for prospective studies that include IBD patients with joint symptoms irrespective of formal SpA classification, and that link immune profiles to tissue inflammation, symptom course, and treatment response.

In conclusion, this study shows that IBD patients without JC have distinct serum immune profiles from axSpA patients, while formal SpA or axSpA diagnosis adds limited additional serum-protein separation among patients who already have joint symptoms. Importantly, IBD patients with JC showed a modest serum immune shift compared with IBD patients without JC, with partial overlap with axSpA-associated proteins. These findings suggest that joint complaints in IBD may mark a biologically relevant subgroup that is not fully captured by current SpA classification. Future longitudinal studies should determine whether this subgroup differs in tissue-level inflammation, clinical course, and response to gut- or joint-directed immunomodulatory therapies.

## Data Availability

Original datasets are available in a publicly accessible repository: 10.5281/zenodo.20491092.

## Supplementary material

Supplementary Figure 1 Comparison of IBD patients without joint complaints (n = 95) to axSpA patients (n = 79). Missing protein values were imputed using half the limit of detection (0.5 × LOD). **(A)** Volcano plot showing 19 proteins are differentially abundant between IBD and axSpA patients. Horizontal dotted line indicates threshold for significance (FDR <0.05) after correction for multiple testing based on the Benjamini-Hochberg procedure. **(B)** Heatmap of 10 proteins with highest MAD with hierarchical clustering (Euclidean distance, complete linkage) shows groups of IBD patients and groups of axSpA patients clustering together. axSpA, axial spondyloarthritis; FDR, false discovery rate; IBD, inflammatory bowel disease; LOD, limit of detection; MAD, median absolute deviation.

Supplementary Figure 2 Clustering of IBD patients without joint complaints (n = 95) to axSpA patients (n = 79). Heatmap of all proteins with hierarchical clustering (Euclidean distance, complete linkage) shows groups of IBD patients and groups of axSpA patients clustering together. axSpA, axial spondyloarthritis; IBD, inflammatory bowel disease.

Supplementary Figure 3 Heatmaps of internal correlation of four protein signatures. Hierarchical clustering was performed using correlation-based distance with complete linkage. Cronbach’s alpha is indicated at the top of each signature’s heatmap.

Supplementary Figure 4 Volcano plots of several group comparisons. **(A)** Comparison of IBD with active (n = 95) versus inactive disease (n = 146). Proteins on the left side of the volcano plot are increased in IBD patients with inactive disease, while proteins on the right side are increased in IBD patients with active disease. Active disease is defined as HBI/SCCAI ≥ 5. **(B)** Comparison of IBD patients without JC (n = 95) to IBD patients meeting SpA classification criteria (n = 58). Proteins on the left side of the volcano plot are increased in IBD patients without JC, while proteins on the right side are increased in a subgroup of the IBD patients with JC who also meet SpA classification criteria. **(C)** Volcano plot showing comparisons of IBD patients without JC (n = 95) to a subgroup of the IBD patients with JC who also have a SpA diagnosis (n = 15). Horizontal dotted lines indicate threshold for significance (FDR <0.05) after correction for multiple testing based on the Benjamini-Hochberg procedure. FDR, false discovery rate; HBI, Harvey-Bradshaw Index; IBD, inflammatory bowel disease; JC, joint complaints; SCCAI, Simple Clinical Colitis Activity Index; SpA, spondyloarthritis.

Supplementary Figure 5 Heatmap of Spearman correlations between serum proteins and disease parameters in JOINT and SPACE cohorts. Spearman correlation coefficients were calculated between serum protein concentrations and disease parameters for JOINT (n = 241, right) and SPACE (n = 151, left) cohorts. Correction for multiple testing was based on the Benjamini-Hochberg procedure, * p <0.05, ** p <0.01.

Supplementary Table 1 Overview of plasma proteins measured with the Olink inflammation panel. List of abbreviations, full protein names, UniProt IDs, limits of detection (LOD), and detection rates for all 92 plasma proteins measured using the Olink Inflammation panel. Proteins excluded from further analysis due to low detection rates are marked grey.

Supplementary Table 2 Overview of differentially abundant proteins and their functions. ^1^West NR, Hegazy AN, Owens BMJ et al. Oncostatin M drives intestinal inflammation and predicts response to tumor necrosis factor-neutralizing therapy in patients with inflammatory bowel disease. Nat Med. 2017 doi: 10.1038/nm.4307. ^2^Morel L, Domingues O, Zimmer J, et al. Revisiting the Role of Neurotrophic Factors in Inflammation. Cells. 2020 Apr 2;9 (4):865. doi: 10.3390/cells9040865. ^3^Bednash JS, Johns F, Patel N, et al. The deubiquitinase STAMBP modulates cytokine secretion through the NLRP3 inflammasome. Cell Signal. 2021 Mar;79:109859. doi: 10.1016/j.cellsig.2020.109859. ^4^Gangoda L, Phan TK, Anand S, et al. Deubiquitinase enzyme STAMBP plays a broad role in both Toll-like and Nod-like receptor mediated inflammation. European Journal of Inflammation. 2020;18. doi:10.1177/2058739220960844. ^5^Tomkinson BE, Brown MC, Ip SH, et al. Soluble CD8 during T cell activation. J Immunol. 1989 Apr 1;142 (7):2230-6. ^6^Sewell AK, Gerth UC, Price DA, et al. Antagonism of cytotoxic T-lymphocyte activation by soluble CD8. Nat Med. 1999 Apr;5 (4):399-404. doi: 10.1038/7398.
