# Supplementary figures and images for: Serum immune profiling suggests overlap between IBD patients with joint complaints and patients with spondyloarthritis
Source: Front Immunol. 2026 Jul 9;17:1827501. doi: 10.3389/fimmu.2026.1827501 (PMC13393219; doi:10.3389/fimmu.2026.1827501)

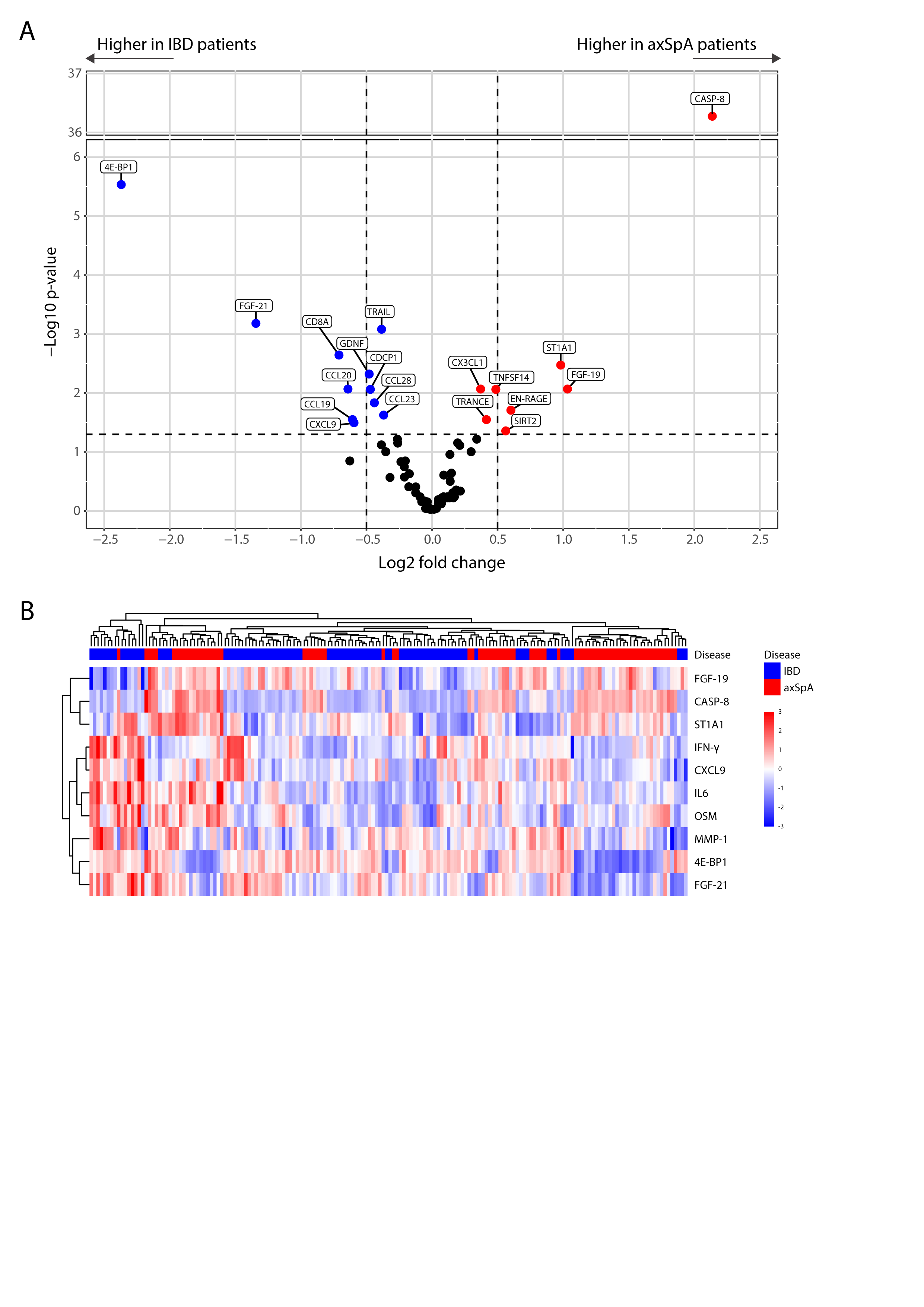

Supplement: Supplementary Figure 1 — Comparison of IBD patients without joint complaints (n = 95) to axSpA patients (n = 79). Missing protein values were imputed using half the limit of detection (0.5 × LOD). (A) Volcano plot showing 19 proteins are differentially abundant between IBD and axSpA patients. Horizontal dotted line indicates threshold for significance (FDR <0.05) after correction for multiple testing based on the Benjamini-Hochberg procedure. (B) Heatmap of 10 proteins with highest MAD with hierarchical clustering (Euclidean distance, complete linkage) shows groups of IBD patients and groups of axSpA patients clustering together. axSpA, axial spondyloarthritis; FDR, false discovery rate; IBD, inflammatory bowel disease; LOD, limit of detection; MAD, median absolute deviation. [file Image1.tif]

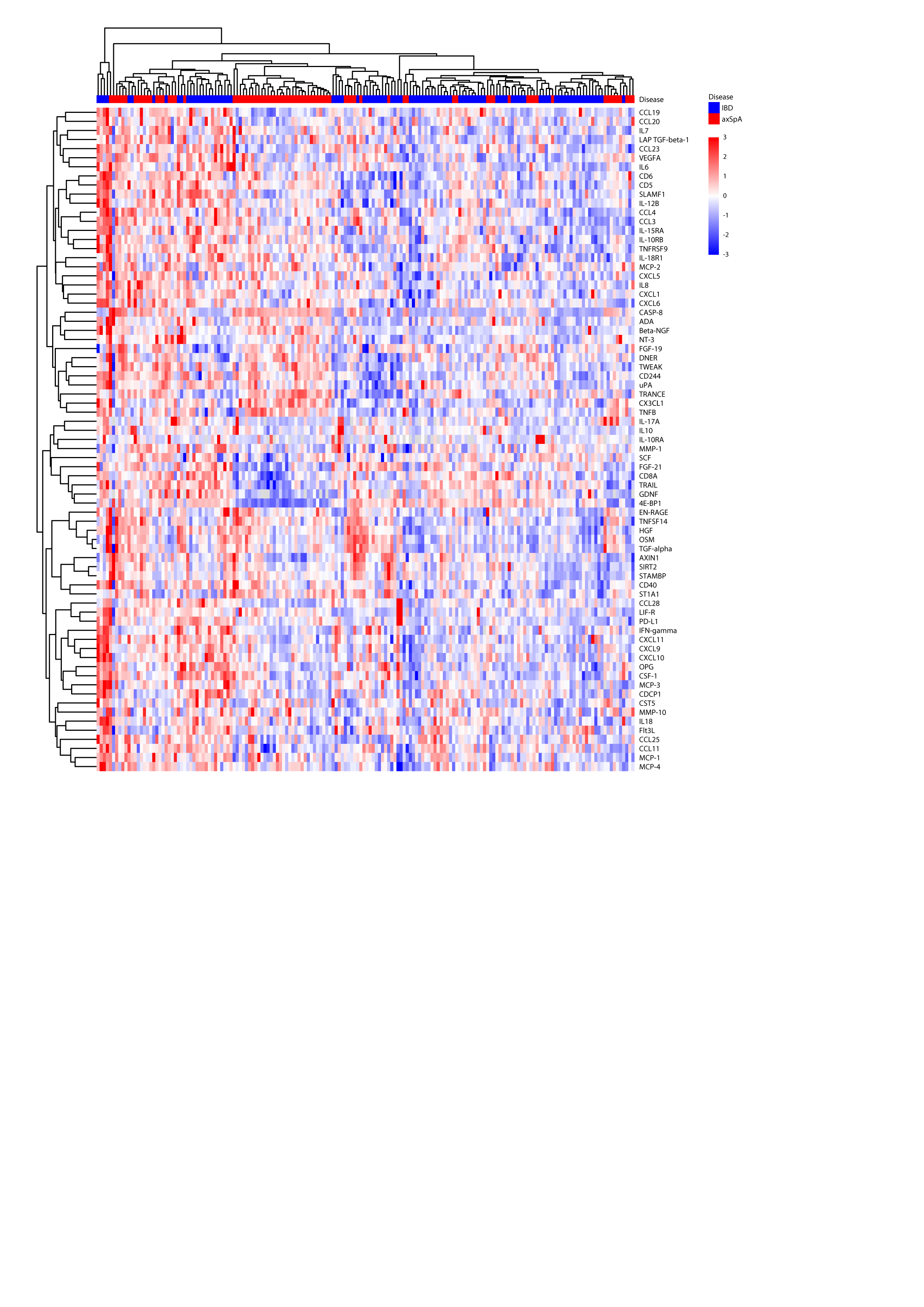

Supplement: Supplementary Figure 2 — Clustering of IBD patients without joint complaints (n = 95) to axSpA patients (n = 79). Heatmap of all proteins with hierarchical clustering (Euclidean distance, complete linkage) shows groups of IBD patients and groups of axSpA patients clustering together. axSpA, axial spondyloarthritis; IBD, inflammatory bowel disease. [file Image2.tif]

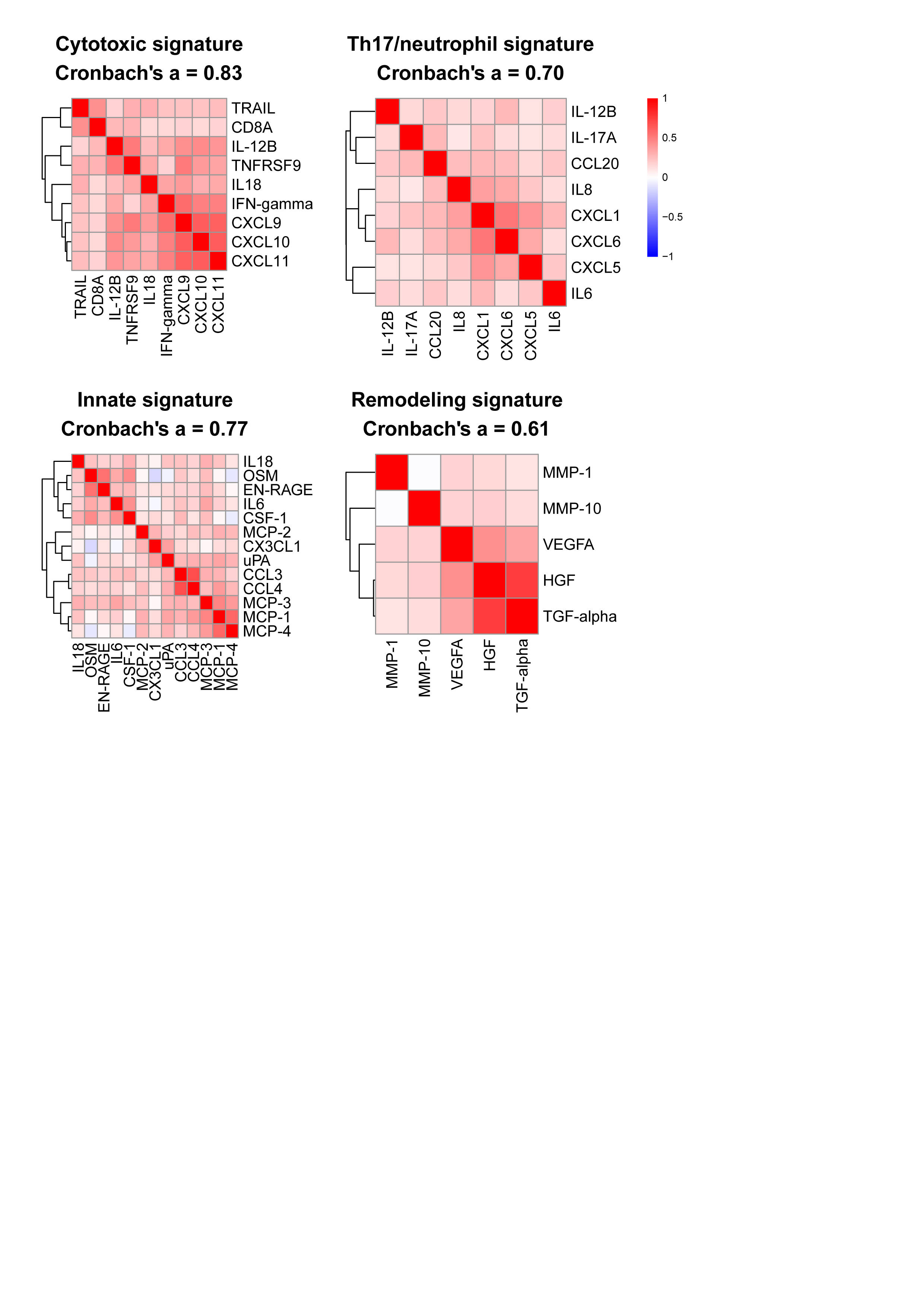

Supplement: Supplementary Figure 3 — Heatmaps of internal correlation of four protein signatures. Hierarchical clustering was performed using correlation-based distance with complete linkage. Cronbach’s alpha is indicated at the top of each signature’s heatmap. [file Image3.tif]

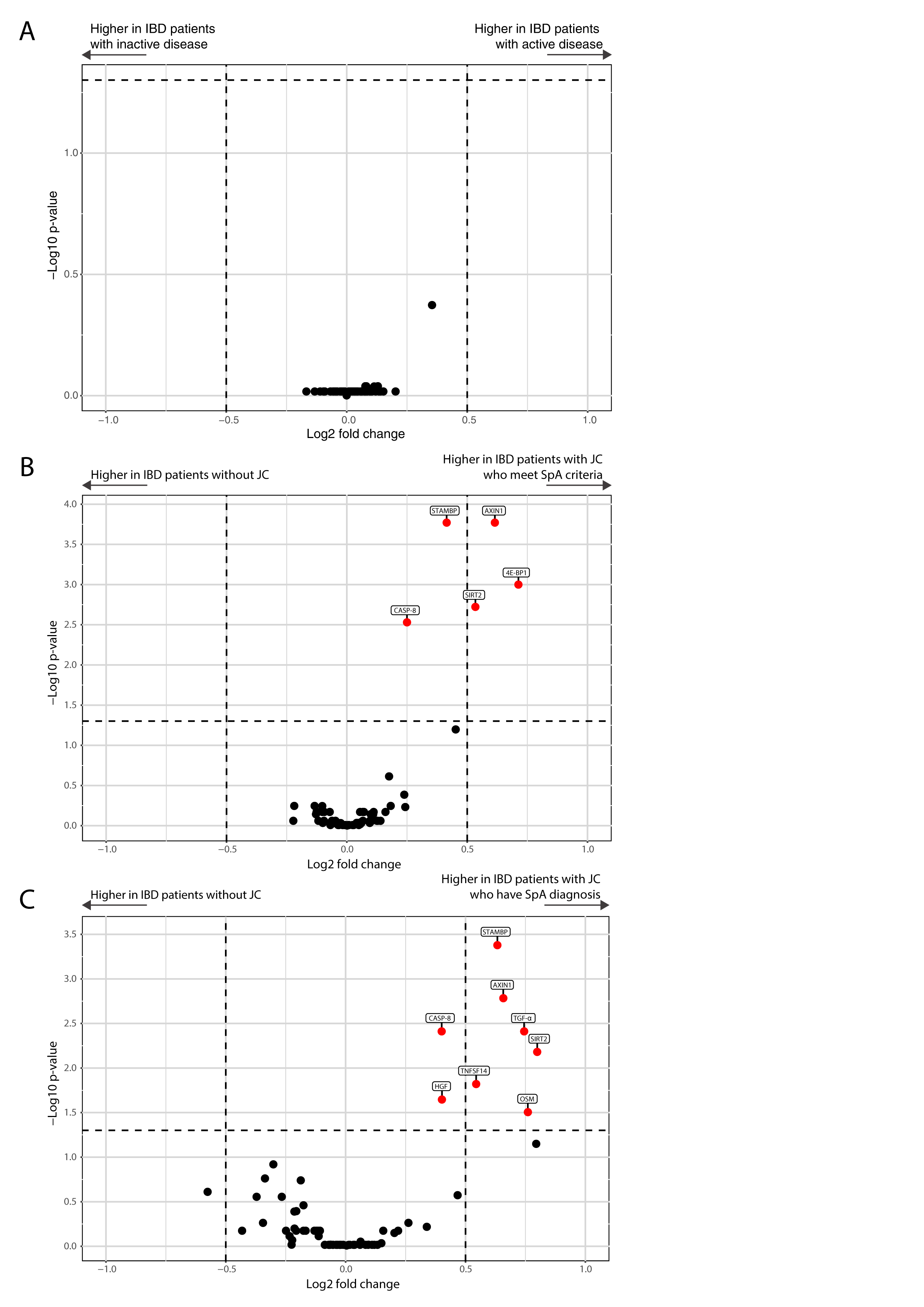

Supplement: Supplementary Figure 4 — Volcano plots of several group comparisons. (A) Comparison of IBD with active (n = 95) versus inactive disease (n = 146). Proteins on the left side of the volcano plot are increased in IBD patients with inactive disease, while proteins on the right side are increased in IBD patients with active disease. Active disease is defined as HBI/SCCAI ≥ 5. (B) Comparison of IBD patients without JC (n = 95) to IBD patients meeting SpA classification criteria (n = 58). Proteins on the left side of the volcano plot are increased in IBD patients without JC, while proteins on the right side are increased in a subgroup of the IBD patients with JC who also meet SpA classification criteria. (C) Volcano plot showing comparisons of IBD patients without JC (n = 95) to a subgroup of the IBD patients with JC who also have a SpA diagnosis (n = 15). Horizontal dotted lines indicate threshold for significance (FDR <0.05) after correction for multiple testing based on the Benjamini-Hochberg procedure. FDR, false discovery rate; HBI, Harvey-Bradshaw Index; IBD, inflammatory bowel disease; JC, joint complaints; SCCAI, Simple Clinical Colitis Activity Index; SpA, spondyloarthritis. [file Image4.tif]

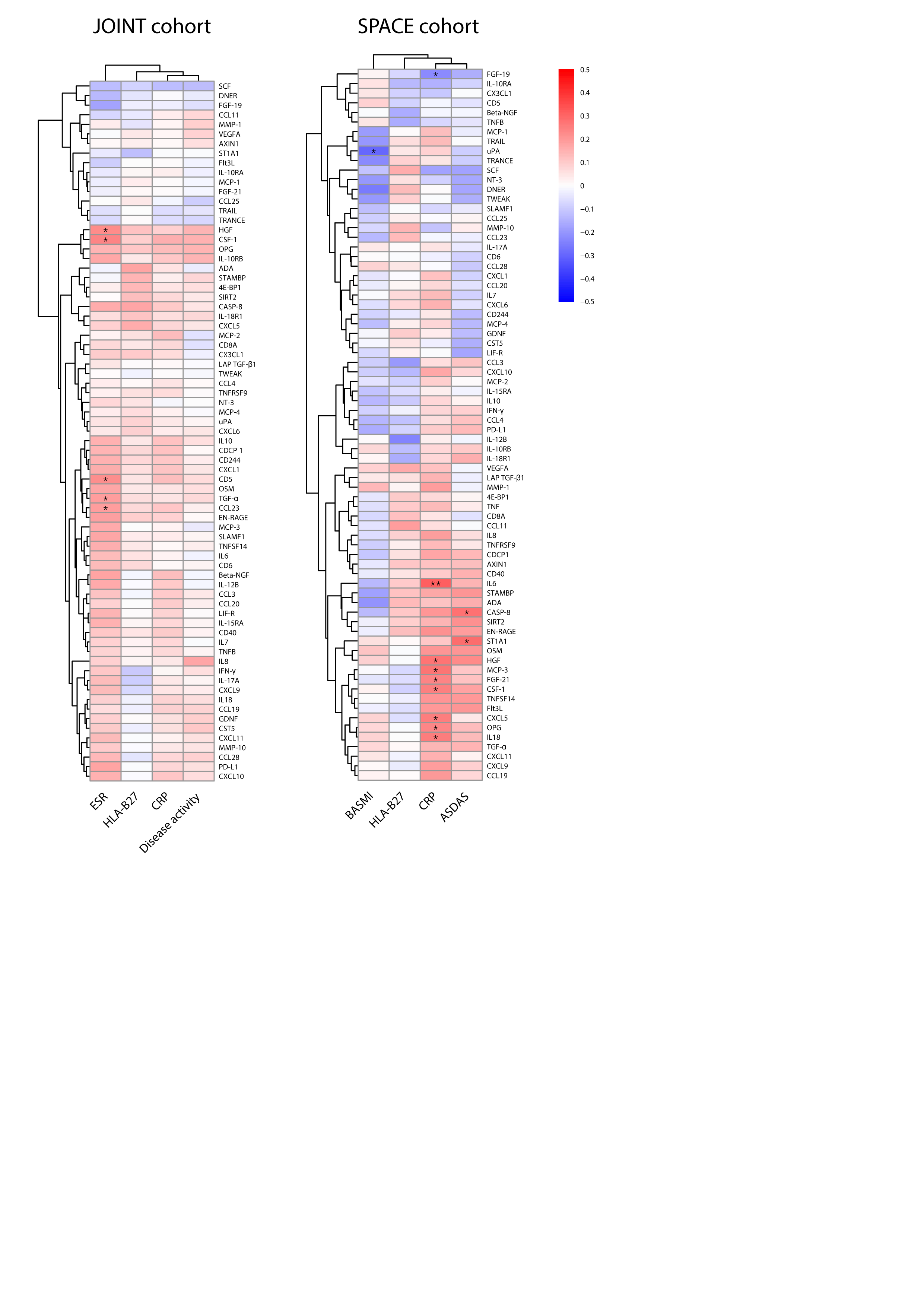

Supplement: Supplementary Figure 5 — Heatmap of Spearman correlations between serum proteins and disease parameters in JOINT and SPACE cohorts. Spearman correlation coefficients were calculated between serum protein concentrations and disease parameters for JOINT (n = 241, right) and SPACE (n = 151, left) cohorts. Correction for multiple testing was based on the Benjamini-Hochberg procedure, * p <0.05, ** p <0.01. [file Image5.tif]
