# Supplementary material for: Serum immune profiling suggests overlap between IBD patients with joint complaints and patients with spondyloarthritis
Source: Front Immunol. 2026 Jul 9;17:1827501. doi: 10.3389/fimmu.2026.1827501 (PMC13393219; doi:10.3389/fimmu.2026.1827501)
Supplement: Supplementary Table 1 — Overview of plasma proteins measured with the Olink inflammation panel. List of abbreviations, full protein names, UniProt IDs, limits of detection (LOD), and detection rates for all 92 plasma proteins measured using the Olink Inflammation panel. Proteins excluded from further analysis due to low detection rates are marked grey. [file Table1.docx]

**Table S1. Overview of plasma proteins measured with the Olink Inflammation panel.**

| **Abbreviation** | **Full name** | **Uniprot ID** | **LOD (NPX)** | **Detection rate in JOINT cohort** | **Detection rate in SPACE cohort** |
| --- | --- | --- | --- | --- | --- |
| 4E-BP1 | Eukaryotic translation initiation factor 4E-binding protein 1 | Q13541 | 1,33093 | 100,0 | 100,0 |
| ADA | Adenosine Deaminase | P00813 | 1,10541 | 100,0 | 100,0 |
| ARTN | Artemin | Q5T4W7 | 0,93223 | 50,6 | 50,3 |
| AXIN1 | Axin-1 | O15169 | 1,03886 | 100,0 | 99,4 |
| Beta-NGF | Beta-nerve growth factor | P01138 | 0,92903 | 100,0 | 100,0 |
| CASP-8 | Caspase-8 | Q14790 | 1,43210 | 100,0 | 100,0 |
| CCL11 | Eotaxin | P51671 | 0,92435 | 100,0 | 100,0 |
| CCL19 | C-C motif chemokine 19 | Q99731 | 1,60901 | 100,0 | 100,0 |
| CCL20 | C-C motif chemokine 20 | P78556 | 2,02941 | 100,0 | 100,0 |
| CCL23 | C-C motif chemokine 23 | P55773 | 1,18353 | 100,0 | 100,0 |
| CCL25 | C-C motif chemokine 25 | O15444 | 0,98738 | 100,0 | 100,0 |
| CCL28 | C-C motif chemokine 28 | Q9NRJ3 | 1,05646 | 100,0 | 100,0 |
| CCL3 | C-C motif chemokine 3 | P10147 | 0,78295 | 100,0 | 100,0 |
| CCL4 | C-C motif chemokine 4 | P13236 | 0,92710 | 100,0 | 100,0 |
| CD244 | Natural killer cell receptor 2B4 | Q9BZW8 | 1,85971 | 100,0 | 100,0 |
| CD40 | CD40L receptor | P25942 | 2,01988 | 100,0 | 100,0 |
| CD5 | T-cell surface glycoprotein CD5 | P06127 | 0,19776 | 100,0 | 100,0 |
| CD6 | T-cell surface glycoprotein CD6 isoform | P30203 | 0,98289 | 100,0 | 100,0 |
| CD8A | T-cell surface glycoprotein CD8 alpha chain | P01732 | 1,30889 | 100,0 | 100,0 |
| CDCP1 | CUB domain-containing protein 1 | Q9H5V8 | 0,52255 | 100,0 | 100,0 |
| CSF-1 | Macrophage colony-stimulating factor 1 | P09603 | 1,06155 | 100,0 | 100,0 |
| CST5 | Cystatin D | P28325 | -0,49513 | 100,0 | 100,0 |
| CX3CL1 | Fractalkine | P78423 | 1,49058 | 100,0 | 100,0 |
| CXCL1 | C-X-C motif chemokine 1 | P09341 | 1,87834 | 100,0 | 100,0 |
| CXCL10 | C-X-C motif chemokine 10 | P02778 | 2,52476 | 100,0 | 100,0 |
| CXCL11 | C-X-C motif chemokine 11 | O14625 | 1,92197 | 100,0 | 100,0 |
| CXCL5 | C-X-C motif chemokine 5 | P42830 | 1,39076 | 100,0 | 100,0 |
| CXCL6 | C-X-C motif chemokine 6 | P80162 | 1,18167 | 100,0 | 100,0 |
| CXCL9 | C-X-C motif chemokine 9 | Q07325 | 1,38212 | 100,0 | 100,0 |
| DNER | Delta and Notch-like epidermal growth factor-related receptor | Q8NFT8 | 1,41733 | 100,0 | 100,0 |
| EN-RAGE | Protein S100-A12 | P80511 | 1,01679 | 100,0 | 100,0 |
| FGF-19 | Fibroblast growth factor 19 | O95750 | 1,18640 | 100,0 | 100,0 |
| FGF-21 | Fibroblast growth factor 21 | Q9NSA1 | 1,51276 | 100,0 | 100,0 |
| FGF-23 | Fibroblast growth factor 23 | Q9GZV9 | 2,48733 | 65,1 | 46,7 |
| FGF-5 | Fibroblast growth factor 5 | P12034 | 1,17192 | 32,0 | 18,8 |
| Flt3L | Fms-related tyrosine kinase 3 ligand | P49771 | 1,78688 | 100,0 | 100,0 |
| GDNF | Glial cell line-derived neurotrophic factor | P39905 | 1,79082 | 98,8 | 81,2 |
| HGF | Hepatocyte growth factor | P14210 | 1,35490 | 100,0 | 100,0 |
| IFN-gamma | Interferon gamma | P01579 | 2,90482 | 99,6 | 100,0 |
| IL-1 alpha | Interleukin-1 alpha | P01583 | 0,10416 | 4,6 | 3,0 |
| IL10 | Interleukin-10 | P22301 | 2,05849 | 99,2 | 100,0 |
| IL-10RA | Interleukin-10 receptor subunit alpha | Q13651 | 0,87677 | 88,0 | 86,7 |
| IL-10RB | Interleukin-10 receptor subunit beta | Q08334 | 1,21703 | 100,0 | 100,0 |
| IL-12B | Interleukin-12 subunit beta | P29460 | 0,27563 | 100,0 | 100,0 |
| IL13 | Interleukin-13 | P35225 | 1,49148 | 27,4 | 38,2 |
| IL-15RA | Interleukin-15 receptor subunit alpha | Q13261 | 0,59574 | 100,0 | 99,4 |
| IL-17A | Interleukin-17A | Q16552 | 1,43340 | 95,0 | 95,2 |
| IL-17C | Interleukin-17C | Q9P0M4 | 1,65470 | 71,0 | 29,7 |
| IL18 | Interleukin-18 | Q14116 | 0,57660 | 100,0 | 100,0 |
| IL-18R1 | Interleukin-18 receptor 1 | Q13478 | 1,65421 | 100,0 | 100,0 |
| IL2 | Interleukin-2 | P60568 | 1,81776 | 1,2 | 0,6 |
| IL-20 | Interleukin-20 | Q9NYY1 | 1,14318 | 8,3 | 7,9 |
| IL-20RA | Interleukin-20 receptor subunit alpha | Q9UHF4 | 1,64960 | 35,3 | 43,6 |
| IL-22 RA1 | Interleukin-22 receptor subunit alpha-1 | Q8N6P7 | 2,85880 | 9,5 | 10,3 |
| IL-24 | Interleukin-24 | Q13007 | 1,71514 | 25,3 | 15,8 |
| IL-2RB | Interleukin-2 receptor subunit beta | P14784 | 1,48048 | 44,8 | 81,2 |
| IL33 | Interleukin-33 | O95760 | 2,03635 | 5,0 | 3,0 |
| IL4 | Interleukin-4 | P05112 | 0,97060 | 21,2 | 31,5 |
| IL5 | Interleukin-5 | P05113 | 1,60232 | 41,5 | 45,5 |
| IL6 | Interleukin-6 | P05231 | 1,70041 | 100,0 | 100,0 |
| IL7 | Interleukin-7 | P13232 | 1,27626 | 100,0 | 100,0 |
| IL8 | Interleukin-8 | P10145 | 1,26484 | 100,0 | 100,0 |
| LAP TGF-beta-1 | Latency-associated peptide transforming growth factor beta-1 | P01137 | 1,32054 | 100,0 | 100,0 |
| LIF | Leukemia inhibitory factor | P15018 | 1,00941 | 10,8 | 12,7 |
| LIF-R | Leukemia inhibitory factor receptor | P42702 | 1,20695 | 100,0 | 100,0 |
| MCP-1 | Monocyte chemotactic protein 1 | P13500 | 1,28502 | 100,0 | 100,0 |
| MCP-2 | Monocyte chemotactic protein 2 | P80075 | 2,04031 | 100,0 | 100,0 |
| MCP-3 | Monocyte chemotactic protein 3 | P80098 | 1,59412 | 98,8 | 98,2 |
| MCP-4 | Monocyte chemotactic protein 4 | Q99616 | 2,06375 | 100,0 | 100,0 |
| MMP-1 | Matrix metalloproteinase-1 | P03956 | 2,27463 | 100,0 | 100,0 |
| MMP-10 | Matrix metalloproteinase-10 | P09238 | 1,70758 | 100,0 | 100,0 |
| NRTN | Neurturin | Q99748 | 1,17581 | 32,4 | 45,5 |
| NT-3 | Neurotrophin-3 | P20783 | 1,42836 | 100,0 | 100,0 |
| OPG | Osteoprotegerin | O00300 | 1,20539 | 100,0 | 100,0 |
| OSM | Oncostatin-M | P13725 | 0,89343 | 100,0 | 100,0 |
| PD-L1 | Programmed cell death 1 ligand 1 | Q9NZQ7 | 2,95419 | 100,0 | 100,0 |
| SCF | Stem cell factor | P21583 | 1,43263 | 100,0 | 100,0 |
| SIRT2 | SIR2-like protein 2 | Q8IXJ6 | 2,06355 | 99,6 | 100,0 |
| SLAMF1 | Signaling lymphocytic activation molecule | Q13291 | 2,01526 | 99,6 | 99,4 |
| ST1A1 | Sulfotransferase 1A1 | P50225 | 1,12675 | 100,0 | 100,0 |
| STAMBP | STAM-binding protein | O95630 | 1,47595 | 100,0 | 100,0 |
| TGF-alpha | Transforming growth factor alpha | P01135 | 1,18028 | 100,0 | 100,0 |
| TNF | Tumor necrosis factor | P01375 | 0,46039 | 99,6 | 100,0 |
| TNFB | TNF-beta | P01374 | 1,27011 | 100,0 | 100,0 |
| TNFRSF9 | Tumor necrosis factor receptor superfamily member 9 | Q07011 | 2,31932 | 100,0 | 100,0 |
| TNFSF14 | Tumor necrosis factor ligand superfamily member 14 | O43557 | 1,03805 | 100,0 | 100,0 |
| TRAIL | TNF-related apoptosis-inducing ligand | P50591 | 0,87868 | 100,0 | 100,0 |
| TRANCE | TNF-related activation-induced cytokine | O14788 | 1,40671 | 100,0 | 100,0 |
| TSLP | Thymic stromal lymphopoietin | Q969D9 | 1,67952 | 19,5 | 21,2 |
| TWEAK | Tumor necrosis factor (Ligand) superfamily, member 12 | O43508 | 0,92688 | 100,0 | 100,0 |
| uPA | Urokinase-type plasminogen activator | P00749 | 1,30010 | 100,0 | 100,0 |
| VEGFA | Vascular endothelial growth factor A | P15692 | 1,81051 | 100,0 | 100,0 |

List of abbreviations, full protein names, UniProt IDs, limits of detection (LOD), and detection rates for all 92 plasma proteins measured using the Olink Inflammation panel. Proteins excluded from further analysis due to low detection rates are marked grey.

**Table S2. Overview of differentially abundant proteins and their functions.**

| **Protein** | **Summary function** |
| --- | --- |
| **Chemoattraction of immune cells** | |
| CCL19 | Chemoattractant for lymphocytes, binds to CCR7. |
| CCL20 | Chemoattractant for helper T-cells, binds to CCR6. |
| CCL23 | Chemoattractant for neutrophils, monocytes, and T-cells. Binds to CCR1. |
| CCL28 | Chemoattractant for eosinophils and T-cells, binds to CCR3 and CCR10. |
| CX3CL1 | Chemoattractant for monocytes and T-cells, binds to CX3CR1. |
| CXCL9 | Chemoattractant for activated T-cells, induced by IFN-γ and mainly produced by macrophages and monocytes. Can also affect movement, growth, and activation state of cells in immune/inflammatory responses. |
| EN-RAGE | Pro-inflammatory protein, produced by neutrophils, monocytes, macrophages, dendritic cells, and epithelial cells. Stimulates cytokine production and leukocyte recruitment. |
| **Neutrophil products** | |
| CDCP1 | Glycoprotein that via its phosphorylation may play a role in negative regulation of cell adhesion and regulation of anchorage versus migration or proliferation versus differentiation. |
| OSM | Cytokine involved in regulation of cytokine production by endothelial cells in inflammation. Also plays a role in osteogenesis, growth regulation and liver development. High OSM expression in the intestine has been associated with anti-TNF treatment resistance in IBD patients.^1^ |
| TNFSF14 | Cytokine that delivers costimulatory signals for activation and proliferation of lymphoid cells. |
| **Cell cycle/apoptosis** | |
| AXIN1 | Can induce apoptosis and is a negative regulator of Wnt signaling. |
| CASP-8 | Most upstream protease of the activation cascade of caspases leading to apoptosis. May be involved in mucosal inflammation in Crohn’s disease by regulating necroptosis of intestinal epithelial cells. |
| SIRT2 | NAD-dependent protein deacetylase, participates in modulation of diverse biological processes, such as cell cycle control, genomic integrity, microtubule dynamics, cell differentiation, metabolic networks, and autophagy. |
| TGF-α | Growth factor that stimulates various biological processes including cell proliferation, differentiation and development. |
| TRAIL | Cytokine that belongs to the tumor necrosis factor family. It can induce apoptosis, and its activity may be modulated by binding to the decoy receptors. |
| **Tissue homeostasis** | |
| FGF-19 | Heparin-binding growth factor, involved in the suppression of bile acid biosynthesis through down-regulation of CYP7A1 expression, following positive regulation of the JNK and ERK1/2 cascades. Stimulates glucose uptake in adipocytes. |
| FGF-21 | Growth factor that stimulates glucose uptake by differentiated adipocytes via induction of glucose transporter SLC2A1/GLUT1 expression. Regulates systemic glucose homeostasis and insulin sensitivity. |
| HGF | Acts as a growth factor for a broad spectrum of tissues and cell types, plays important roles in angiogenesis, tumorigenesis, and tissue regeneration. |
| ST1A1 | Sulfotransferase produced by enterocytes, paneth cells, and hepatocytes. It catalyzes conjugation of sulfate to many hormones, drugs, and xenobiotic compounds. |
| **Inflammation mediation** | |
| 4E-BP1 | Repressor of translation initiation that mediates the regulation of protein translation by hormones, growth factors and other stimuli that signal through the MAP kinase and mTORC1 pathways. |
| GDNF | Neurotropic factor that can bind to various TGF-beta receptors and promotes cell survival and differentiation. Plays an anti‑inflammatory role in the gut by downregulating the expression of pro-inflammatory cytokines and upregulating the expression of tight junction proteins.^2^ |
| IL-10RA | Receptor for IL-10, plays a role in anti-inflammatory signal transduction. |
| STAMBP | Deubiquitinase with both pro- and anti-inflammatory effects through regulation of inflammosomes.^3,4^ Also plays a role in cell growth, and can potentiate BMP signaling. |
| **Cytotoxic T-cell activation** | |
| CD8A | Exists in membrane-bound and soluble forms. Soluble CD8a is secreted by cytotoxic T-cells upon activation, and can in turn antagonize cytotoxic T-cell activation.^5,6^ |
| **Bone metabolism, immune regulation** | |
| TRANCE | Also called RANKL or TNFSF11, plays a role in bone metabolism. Als plays a role in dendritic cell maturation, is expressed by T‑cells. |

^1^West NR, Hegazy AN, Owens BMJ et al. Oncostatin M drives intestinal inflammation and predicts response to tumor necrosis factor-neutralizing therapy in patients with inflammatory bowel disease. Nat Med. 2017 doi: 10.1038/nm.4307. ^2^Morel L, Domingues O, Zimmer J, et al. Revisiting the Role of Neurotrophic Factors in Inflammation. Cells. 2020 Apr 2;9(4):865. doi: 10.3390/cells9040865. ^3^Bednash JS, Johns F, Patel N, et al. The deubiquitinase STAMBP modulates cytokine secretion through the NLRP3 inflammasome. Cell Signal. 2021 Mar;79:109859. doi: 10.1016/j.cellsig.2020.109859. ^4^Gangoda L, Phan TK, Anand S, et al. Deubiquitinase enzyme STAMBP plays a broad role in both Toll-like and Nod-like receptor mediated inflammation. European Journal of Inflammation. 2020;18. doi:10.1177/2058739220960844. ^5^Tomkinson BE, Brown MC, Ip SH, et al. Soluble CD8 during T cell activation. J Immunol. 1989 Apr 1;142(7):2230-6. ^6^Sewell AK, Gerth UC, Price DA, et al. Antagonism of cytotoxic T-lymphocyte activation by soluble CD8. Nat Med. 1999 Apr;5(4):399-404. doi: 10.1038/7398.
